# Ketogenic and Low FODMAP Diet in Therapeutic Management of a Young Autistic Patient with Epilepsy and Dysmetabolism Poorly Responsive to Therapies: Clinical Response and Effects of Intestinal Microbiota

**DOI:** 10.3390/ijms23158829

**Published:** 2022-08-08

**Authors:** Alexander Bertuccioli, Marco Cardinali, Francesco Di Pierro, Giordano Bruno Zonzini, Maria Rosaria Matera

**Affiliations:** 1 Department of Biomolecular Sciences, University of Urbino Carlo Bo, 61122 Urbino, Italy; 2 Department of Internal Medicine, Infermi Hospital, AUSL Romagna, 47921 Rimini, Italy; 3 Digestive Endoscopy Unit and Gastroenterology, Fondazione Poliambulanza, 25124 Brescia, Italy; 4 Scientific & Research Department, Velleja Research, 20125 Milano, Italy; 5 Department of Pediatrics and Pediatric First Aid Della Misericordia Hospital, Usl Toscana Sud Est, 58100 Grosseto, Italy

**Keywords:** microbiota, ketogenic diet, FODMAPs, seizures, ASD, ASD-epilepsy, case report

## Abstract

Autism spectrum disorder (ASD) is often associated with several intestinal and/or metabolic disorders as well as neurological manifestations such as epilepsy (ASD-E). Those presenting these neuropathological conditions share common aspects in terms of gut microbiota composition. The use of microbiota intervention strategies may be an approach to consider in the management of these cases. We describe the case of a 17-year-old girl affected by ASD, reduced growth, neurological development delay, mutations in the PGM1 and EEF1A2 genes (in the absence of clinically manifested disease) and, intestinal disorders such as abdominal pain and diarrhea associated with weight loss. As she demonstrated poor responsiveness to the therapies provided, we attempted two specific dietary patterns: a ketogenic diet, followed by a low fermentable oligosaccharides, disaccharides, monosaccharides and polyols (FODMAP) diet, with the aim of improving her neurological, metabolic, and intestinal symptoms through modulation of the gut microbiota’s composition. The ketogenic diet (KD) provided a reduction in *Firmicutes*, *Bacteroidetes*, and *Proteobacteria*. Although her intestinal symptoms improved, KD was poorly tolerated. On the other hand, the passage to a low FODMAPs diet produced a significant improvement in all neurological, intestinal, and metabolic symptoms and was well-tolerated. The following gut microbiota analysis showed reductions in Actinobacteria, *Firmicutes*, *Lactobacilli*, and *Bifidobacteria*. The alpha biodiversity was consistently increased and the *Firmicutes*/*Bacteroidetes* ratio decreased, reducing the extent of fermentative dysbiosis. Gut microbiota could be a therapeutic target to improve ASD-related symptoms. Further studies are needed to better understand the correlation between gut microbiota composition and ASD, and its possible involvement in the physiopathology of ASD.

## 1. Introduction

Autism spectrum disorder (ASD) is a condition that can be associated with other neurological manifestations such as epilepsy, defining a phenotypic manifestation common to various genetic disorders: ASD-epilepsy (ASD-E) [1]. This clinical manifestation can be considerably worsened by associated metabolic disorders, which can make patient management a decidedly complex task, especially when a known clinical–syndromic scenario is not readily recognizable. A low fermentable oligosaccharides, disaccharides, monosaccharides and polyols (FODMAP) diet was proposed by Nogay et al. as a possible intervention for the management of gastrointestinal disorders in children with ASD-E. This diet begins with the assumption that insufficient digestion and absorption of some short-chain carbohydrates can lead to symptoms such as abdominal pain and distension, diarrhea and/or intestinal constipation, which also occur at the microbiota level. In general, the initial benefits are observed after the first week of the diet, and a period of between 2 and 6 weeks, at least, is recommended to evaluate the clinical response [2].

## 2. Methods and Aim of the Case Report

The case presentation was redacted according to CARE guidelines [3]. The aim of this report was to describe how simple interventions such as a low-FODMAP diet, through shaping of the gut microbiota, improved ASD-E-related symptoms along with intestinal and metabolic symptoms in a 17-year-old Caucasian girl presenting with a very complex clinical pattern.

## 3. Case Presentation

Here, we describe the case of a 17-year-old Caucasian girl with a history of generalized seizures (atypical atonic crises) with an early onset in infancy, associated with neurological development delay corresponding to autism spectrum disorder and reduced growth. The girl also suffered stypsis and frequent episodes of abdominal pain. Genetic examination revealed a mutation in the phosphoglucomutase 1 (PGM1) gene, which is responsible for a rare congenital genetic disease affecting glycosylation and glycogen storage [4], and a variant of the eukaryotic translation elongation factor 1 alpha 2 (EEF1A2) gene on the long arm of the cr 20, which is responsible for unspecified early-onset epileptic encephalopathy and autosomal dominant non-syndromic intellectual disability [5] in the absence of clinically manifested disease. Other chromosomal, genetic, and mitochondrial causes were excluded during thorough examinations in important European pediatric hospitals. Among the different therapeutic approaches to managing epilepsy, vigabatrin, valproic acid and pyridoxine, carbamazepine, clonazepam, and clobazan had been attempted over time, resulting in a limited neurological benefit for a short period of time, especially with valproic acid and pyridoxine and, subsequently, clonazepam; however, her behavior and interaction worsened. Additionally, she had worsened abdominal pain and stool consistency, potentially due to the involvement of the microbiota as reported by Ilhan et al. [6]. Adverse intestinal reactions particularly seemed to occur following the intake of a large number of pharmacological excipients in a similar way to that described for carboxymethylcellulose [7]. However, drugs are rarely available in pharmaceutical form without or with a reduced content of excipients, and resolving these symptoms could prove very complex. Since her first days of life, the patient had suffered from hypoglycemic crises exacerbated by the introduction of fruit and carbohydrates during complementary feeding, and she had been documented with a glycemic holter at the age of 22 months. The incidence of these events reduced after increasing meal frequency and adding corn derivatives to her diet; a reduction in hypoglycemic episodes was also noted following valproic acid suspension. Hyperinsulinism was excluded by laboratory and radiological exams. Further glycemic stabilization was obtained after introducing acarbose. At the age of 15, the recurrence of frequent episodes of generalized seizures required the introduction of clonazepam; a new onset of diarrheic disorder with poorly consistent yellowish and greenish stools was also noted, associated with weight loss. Further laboratory exams showed the absence of Rotavirus, Campylobacter, Shigella, Salmonella, and Yersinia in the stools, while the complete blood count, cholesterol, total proteins, iron, C-reactive protein, and electrolyte exams were normal, virtually excluding malabsorption and/or inflammatory bowel diseases. 

### 3.1. Microbiota Analysis: A Possible Turning Point?

In March 2021, a microbiota analysis was performed according to the method used by Mancabelli and Milani [8] (by MyMicrobiota Lab, Pontenure, PC, Italy). A fermentative dysbiosis pattern was observed, with Firmicutes dominance, Actinobacteria overgrowth, and high presence of lactate-producing bacteria such as Bifidobacteria, Lactobacillus, and Streptococcus; high lactate production was balanced by the higher presence of Negativicutes. This pattern is not surprising [9], as it has been observed in similar cases of drug-resistant epilepsy. After these findings, a short treatment with rifaximin was proposed [10], followed by the administration of a supplement with *Streptococcus salivarius* K12 [11] at 1 billion UFC (notified to the Italian Ministry of Health as a food supplement by Pharmextracta SpA, Pontenure, PC, Italy, complying with law No. 196-2004, notification number: 53435; marketed in Italy under the name of Bactoblis) once a day, *Berberis vulgaris* (255 mg), hydrolyzed Guam gum (750 mg), and melatonin (0.5 mg) [12] (notified to the Italian Ministry of Health as a food supplement by Pharmextracta SpA, Pontenure, PC, Italy, complying with law No. 196-2004, notification number: 12; marketed in Italy under the name of Dibiesse) two times a day, with rapid resolution of diarrhea, mood, weight recovery, and daily activities (with all the limitations of the case). The decision to administer a nutraceutical specifically formulated for the management of diarrhea [12] was made, in addition to the approach based on rifaximin followed by probiotics (already described in the literature [10]) to provide rapid clinical benefit to the patient, with the aim of improving her quality of life as soon as possible.

### 3.2. Dietary Approaches following Stabilization of the Intestinal Framework

**Figure 1:**
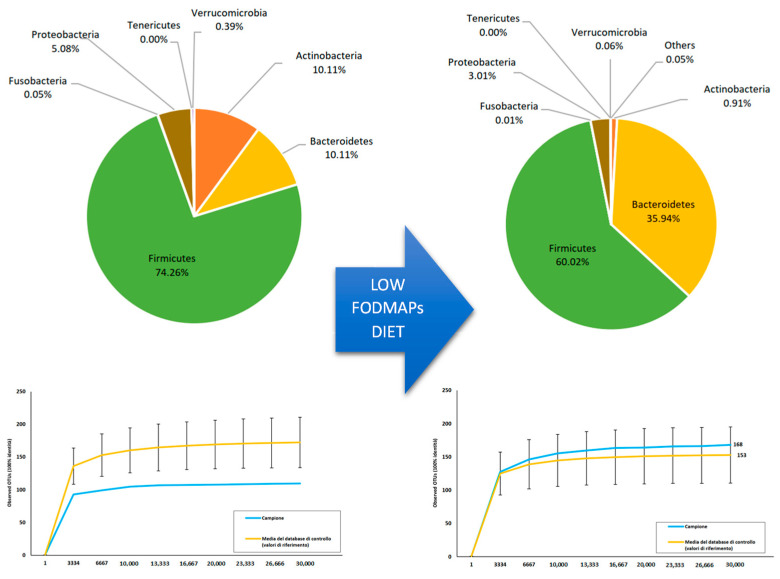
Variation in taxonomic composition and biodiversity following a low-FODMAP diet.

Following this result, a ketogenic diet approach was administered with the aim of reducing the dominance of Firmicutes reduction, and Bacteroidetes and Proteobacteria growth in accordance with Zhang et al. [13]. In the following months, a ketogenic diet with a 1.95–2.30 k-ratio was poorly tolerated, as frequent hypoglycemic events occurred. With a progressive k-ratio and ketone reduction, hypoglycemic events improved but notable asthenia was noted. However, a further modest improvement in intestinal symptoms was reported, as expected. Given these results, a low-FODMAP diet was then started [10]; after a few weeks, the patient’s condition improved with glycemic steadiness, a further normalization of bowel dynamics, and less frequent and severe episodes of seizures. In January 2022, the microbiota analysis was repeated: the alpha biodiversity was consistently increased. The presence of Firmicutes and Actinobacteria was drastically reduced, with a relevant increase in Bacteroidetes. As a result, the Firmicutes/Bacteroidetes ratio decreased, resulting in a lesser extent of fermentative dysbiosis. Additionally, lactate-producing bacteria such as Lactobacilli and Bifidobacteria were markedly reduced, as shown in Figure 1.

## 4. Discussion

The management and analysis of this case proved to be very complex, especially considering that it has not yet been possible to identify a single common pathological element or a well-defined syndrome that would allow for the planning of a specific treatment. In the administration of the different treatments, with the aim of clinical management of the symptoms, particular attention was paid to the possibility that elements such as pro-inflammatory cytokines, amino acids, endocrine neurotransmitters, short-chain fatty acids (SCFA), free radicals of oxygen (ROS), and other metabolites produced in the intestine may be basis of neuro-mediated stimuli and alterations in the permeability of the mucosal barrier, acting dysfunctionally at the immune, inflammatory, and blood–brain barrier (BBB) permeability levels. This potentially favors a condition of neuroinflammation, a situation described as potentially being able to further contribute to intestinal dysbiosis in a two-way manner [14].

### 4.1. The Possible Role of the Microbiota

In this context, increased levels of *Firmicutes* (at the expense of reduced levels of *Bacteroidetes*) have been described in epilepsy cases with consequent reduced activity in the threonine, tryptophan, and creatinine pathways. Additionally, high levels of *Clostridium* and *Lactobacillus* with an altered *Firmicutes*/*Bacteroidetes* ratio have been described in ASD, resulting in alterations in the tricarboxylic pathway (↑ succinic acid, ↓ citric acid), the kynurenine pathway (↑ xanthurenic acid and quinolinic acid, ↓ kynurenic acid), and serotonin pathways (↓ melatonin); increases in the bacterial degradation of tryptophan; increases in purine catabolites, glutamate, aspartate, and 3-aminoisobutyric and glutaric acid; and decreases in glutathione, creatinine, and isoleucine [13]. In this regard, the ketogenic diet could prove to be a potentially interesting tool: as described by Zhang et al. [13], following 6 months of therapy with a ketogenic diet, in one sample, a lower α-diversity was found in epileptic children with a reduction in *Firmicutes* levels and an increase in *Bacteroidetes* levels. Furthermore, Xie et al. described how the intestinal microbiota of epileptic infants is significantly more rich in pathogenic bacteria compared with that of healthy controls, showing that significant changes could be observed after a week of following a ketogenic diet [15]. These elements become particularly significant if examined in light of the correlation between elevated levels of Bacteroidetes and cognitive performance [16,17].

### 4.2. Ketogenic Diet and Microbiota

The patient did not tolerate a ketogenic diet well, an interesting fact that could be interpreted on the basis of what was reported by Tagliabue et al. [18]. In the microbiota of children with GLUT1 deficiency syndrome following a ketogenic diet for 3 months, a significant increase in *Desulfovibrio* spp. was reported, similar to in our patient, as this bacterial group is believed to be involved in the exacerbation of the inflammatory condition of the intestinal mucosa. This suggests that the extent of these interventions is still yet to be understood, as the relationship between the effects was also directly related to the ketogenic diet and the changes generated in the intestinal microbiota [19]. It is important to underline what was reported by Roussin et al. According to the literature review, *Desulfovibrio* is present in at least 50% of children with ASD; other characteristic aspects described by various authors are the increase in Clostridium, the reduction in Bifidobacterium, and the increase in *Faecalibacterium* [17], aspects that were not initially observable in our patient but were outlined following improvements in the dynamics of her bowel habits.

### 4.3. Low-FODMAP Diet and Microbiota: Microbiota Pattern Change as a Possible Explanation?

The improvement found with the low-FODMAP diet—which, unlike the ketogenic diet, was well-tolerated—could also be analyzed on the basis of what was observed in patients suffering from propionic acidosis. This is a genetic disorder characterized by propionate accumulation, which manifests as a delay in neurological development, a high prevalence of ASD (21%) [20], and mitochondrial dysfunction associated with significant oxidative stress [21]. It is tempting to say that propionic acid, which is also a product of bacterial metabolism efficiently obtained from fermentable substances such as FODMAPs, could play a very important role in similar neurological and systemic diseases. The success of the low-FODMAP diet could also help explain the adverse intestinal reaction to some drug excipients, which were possibly used as an energy substrate for fermentation processes by intestinal bacteria [7]. Indeed, Vervevier et al. described cases of inflammatory bowel disease (IBS) characterized by a pathogenic gut microbial signature, enriched in *Firmicutes* and genes for carbohydrate and amino acid metabolism, but depleted in *Bacteroidetes* species. This microbiota composition switched towards a pattern similar to the control group following a low-FODMAP diet [22], in a very similar way to what was found in our case, where following a low-FODMAP diet and, subsequently, normalization of bowel movements, a microbial pattern much more similar to those described in similar patients emerged. Additionally, what was reported by Lindefeldt et al. should be considered. Analysis of the microbiota of 12 children with drug-resistant epilepsy who were administered a ketogenic diet, highlighted a reduction in *Bifidobacterium* and an increase in *Escherichia*—as in our case—as relevant elements that could induce functional changes [23] while enhancing the effects on the microbiota due to the reduced consumption of fiber, which, in our case and in the work of Vervevier et al. [22], have been associated with positive effects.

### 4.4. Gut Microbiota Transplants: Could This Represent the Next Step?

To confirm the potential role played by the microbiota in epilepsy, it is interesting to highlight the case described by He et al. involving a patient suffering from Crohn’s disease, epilepsy, and continuous menstrual irregularities who, following a fecal transplant, reported complete clinical remission of epileptic seizures, allowing the suspension of therapy, with remission maintained until the end of the 20-month follow-up. The patient showed a continuous improvement in quality of life, she started to work, her menstrual cycle was regularized at 6 weeks with a normal flow, and she was able to conceive a child with eutocic delivery [24]. These results underline that there are still numerous pathophysiological aspects to be understood in the host–microbiota relationship, even in apparently unrelated complex situations.

## 5. Conclusions

The analysis of this case shows that there are still many pathophysiological aspects to consider, both in the management of complex cases and possibly also in the management of ordinary cases, integrating the potential of clinical intervention with respect to what has been achieved so far. Future studies will be needed to understand whether the benefits deriving from the ketogenic diet (when tolerated) refer exclusively to metabolic aspects, to remodulation of the intestinal microbiota, or both. A great challenge in the coming years will probably be understanding the role of nutrition in the modulation of the microbiota, considering, as in the case described, that foods rich in fiber—generally considered healthy—and bacteria—considered exclusively protective—could also exert a dysfunctional role instead. A low-FODMAP diet combined with appropriate pharmacological, nutraceutical, and bacterial precision therapy derived from an in-depth study of the intestinal microbiota facilitated significant clinical benefits capable of significantly impacting patients’ health and quality of life. Expanding this knowledge will probably lead to a review of some concepts, such as that of a healthy diet and protective bacteria, which are taken for granted and consolidated today, leading us to understand how, most likely, there is no diet or “healthy” microbial pattern valid for all situations. Understanding of these factors may clarify, in the future, what role the different techniques available for modulation of the intestinal microbiota may have for better clinical management, even of very complex cases such as the one described.

## Data Availability

The data presented in this study are available on request from the corresponding author.
